# The ambition for a one-and-done vision-saving AAV vector

**DOI:** 10.1016/j.omtm.2025.101414

**Published:** 2025-02-03

**Authors:** Therese Cronin

**Affiliations:** 1 Nantes Université, CHU Nantes, INSERM, UMR1089, 44000 Nantes, France

## Main text

In 2006, Regeneron Pharmaceuticals signed a deal with Bayer to test a compound developed in the cancer field on proliferative eye diseases. This compound, aflibercept, is a soluble recombinant fusion protein composed of the binding domain 2 of the vascular endothelial growth factor receptor 1 (VEGFR1) fused using the Fc region of human immunoglobulin G1 to domain 3 of VEGFR2. Aflibercept acts as a decoy to inhibit activation of the VEGFR, thus limiting the unchecked proliferation of leaky capillaries, as occurs in the vascular compartment of the eye, the choroid. This choroidal neovascularization (CNV) is a hallmark of the “wet” form of age-related macular degeneration (*wet*AMD) and can lead to choroidal membrane rupture and, ultimately, vision loss. The aflibercept decoy, or “trap,” could block and potentially reverse this neovascularization, reverting *wet*AMD to a stationary disease.

The pivotal VIEW1 and VIEW2 clinical trials from 2007 to 2011 tested this strategy and found that a remarkable 90% of treated *wet*AMD patients maintained visual acuity 1 year after treatment. In these trials aflibercept was compared with ranibizumab (Lucentis, Genentech/Roche), one of several aflibercept alternatives that include monoclonal antibodies and tyrosine kinase inhibitors, which block VEGF directly or inhibit the activation of its receptors. Collectively known as anti-VEGFs, these compounds are already one of the biggest successes of ocular therapies, with significant potential due to the staggering number of patients who would continue to benefit from this treatment. The global prevalence of AMD was estimated at 8.7% in 2020, a percentage that will inevitably increase in the coming decades.^1^ While the neovascular form represents just 10%–15% of AMD diagnoses, due to its severity it accounts for over 90% of AMD-linked clinical blindness. In the years since the VIEW trials, Eylea, the aflibercept brand name, has also been approved for macular edema secondary to central retinal vein occlusion and branch retinal vein occlusion, diabetic macular edema, diabetic retinopathy, and myopic CNV.

The anti-VEGF therapies have been a massive boon to the ophthalmology clinic and to a world with a rapidly aging demographic, but there are downsides. Repeated ocular injections from every 4 to 8 weeks are necessary to ensure sufficient concentration of the compound to offset the growth of new leaky capillaries under the choroid. Encouragingly, recent trials prove that increasing the concentration of product injected from 2 to 8 mg allows the spacing out of injections to up to 12 weeks, leading to marketing authorization of the high-dose product in the United States in 2023 and in the European Union in 2024.^2^ This repetitive injection regimen is nonetheless a significant demand on ophthalmologists’ time and makes a successful outcome in a vulnerable demographic riskier, with individuals missing appointments due to illness or other unforeseen factors. A striking example is the increase in global vision loss due to missed appointments during the COVID-19 pandemic.^3^^,^^4^

Given these limitations, no sooner had anti-VEGF become the standard of care for *wet*AMD than the race was on to couple it with the other great success story from blindness research, the pioneering use of adeno-associated virus (AAV) in retinal gene therapy. The ambition for a gene therapy that would block CNV had previously been put to the test using a soluble form of the VEGF receptor sFlt-1, expressed by AAV. However, its efficacy was insufficient to be used as a one-shot treatment, and therefore is currently proposed as a therapy in conjunction with pharmacological anti-VEGF injections.^5^

An AAV expressing an anti-VEGF that can avoid use of the recombinant protein altogether has since led to a number of clinical trials of AAVs carrying variations on an aflibercept transgene (these studies were registered at ClinicalTrials.gov: NCT05197270, NCT05536973, NCT04704921, and NCT05407636). With differing transgene elements, regulatory components, serotypes, doses, and routes of delivery, the value of these vectors depends on having efficacy in line with the standard treatment of repeated aflibercept injections. Of course, safety is also key. While recombinant AAV has proven its value for safe transgene expression at moderate doses in the retina, certain serotypes have shown dose-dependent adverse events.^6^^,^^7^ In addition to the dose, an adverse outcome is closely linked to the underlying pathology, and an aging patient group may be more vulnerable to such innate inflammatory responses. Therefore, it is essential to have a highly efficient vector that can achieve sufficient sustained secretion of aflibercept from the transduced cells while remaining within the limits of a non-toxic vector dose. This is the challenge addressed by Shim et al. with NG101.^8^ There are three aspects in which their formulation balances efficacy and safety. First, the authors optimized a short promoter, called CAT311, to ensure enhanced and robust transgene expression from an AAV8 vector. Second, *cis*-sequences present on the transgene cassette were designed to potentially offset inflammatory responses. The single-hybrid intron immediately 5′ to the coding sequence carries target sites of miR-142-3p, a microRNA specific to antigen-presenting cells (APCs).^9^ It is hypothesized, although not shown in the paper, that the presence of these sequences ensures that transgene expression is dampened in AAV-transduced APCs and thus minimizes any host immune response specific to aflibercept expression. Since miR-142 is broadly conserved between species, the protective effects observed here in mice and macaques may be expected to extend to humans. Third, while the injections of recombinant aflibercept in the form of Eylea are intravitreal, a subretinal injection is preferred for NG101. This surgical procedure is considerably more disruptive than an intravitreal injection, but it ensures a locally delivered dose of the vector to the affected macular region, thereby limiting the overall amount of vector and avoiding the more immunogenic intravitreal compartment.

The *in vivo* results are encouraging. In mice, despite the low dosage, the 1E6 vector genomes (vg)/eye delivered by subretinal injection resulted in aflibercept concentrations in whole-eye lysates that were equivalent to the tested intravitreally injected Eylea dose. Therapeutic efficacy was similarly matched through measures of reduced leakage from choroidal vasculature and reduced retinal atrophy. In macaques, the dose of 3E9 vg/eye was sufficient to inhibit a laser-induced CNV formation and was found to show durable expression up to 1 year after injection. The only current AAV clinical trial for *wet*AMD matching this dose is the lowest dosing cohort of the AAV8-based RGX-314 formulation, which delivers a monoclonal antibody fragment.^10^

Based on the promising preclinical data presented in this study, a phase 1/2a clinical trial to evaluate the safety and preliminary therapeutic efficacy of NG101 in *wet*AMD patients (this study was registered at ClinicalTrials.gov: NCT05984927) has been added to the list of trials testing anti-VEGF ocular therapy. This is a very good thing. New pioneering formulations must reach a high bar before disrupting an already revolutionary standard of care. Shim et al. provide us with another promising one-and-done, vision-saving candidate.

## Declaration of interests

The authors declare no competing interests.
